# Influence of Lipid Matrix Composition on the Intestinal Permeation of Curcumin-Loaded Lipid Nanoparticles

**DOI:** 10.3390/pharmaceutics18081024

**Published:** 2026-08-18

**Authors:** Anam Sajjad Khan, Daniela Müller, Cornelia M. Keck

**Affiliations:** Department of Pharmaceutics and Biopharmaceutics, Philipps-Universität Marburg, Robert-Koch-Str. 4, 35037 Marburg, Germany; anam.khan@pharmazie.uni-marburg.de

**Keywords:** lipid nanoparticles, nanostructured lipid carriers, lipid matrix composition, intestinal permeation efficacy, ex vivo porcine gut model

## Abstract

**Background:** Lipid nanoparticles are widely investigated as oral drug delivery systems, but their intestinal performance remains difficult to predict based only on physicochemical properties. This study aimed to elucidate how the lipid matrix composition influences the intestinal permeation of curcumin from lipid nanoparticles. **Methods:** Curcumin-loaded nanoemulsions, nanostructured lipid carriers with defined solid-to-liquid lipid ratios, and solid lipid nanoparticles were prepared by high-pressure homogenization. All formulations were characterized with respect to particle size, polydispersity index, and zeta potential before and after simulated intestinal pre-incubation in a simplified SDS-containing intestinal fluid. Intestinal permeation was evaluated ex vivo using porcine gut tissue by analysis of semi-quantitative fluorescence-based permeation readouts (ART) and mean permeation depth (MPD) after 30 and 60 min. **Results:** All formulations maintained stable physicochemical properties with particle sizes around 200 nm and negative zeta potentials; pre-incubation increased the negativity of the zeta potential but left particle size unchanged. Despite similar attributes, the formulations differed in intestinal curcumin permeation based on time and composition. At 30 min, nanoemulsions and mixed nanostructured lipid carriers achieved the highest performance. By 60 min, lipid carriers with more liquid lipid significantly increased both the fluorescence intensity and the depth of curcumin permeation, while other systems showed little further improvement. **Conclusions:** The intestinal permeation of drug from lipid nanoparticles is governed by the lipid matrix architecture and its interaction with the hydrated intestinal environment, which together affect drug-release kinetics and the ability to sustain a trans-epithelial concentration gradient over time. Thus, optimizing oral lipid nanoparticles requires time-resolved, biologically relevant models rather than physicochemical characterization alone, consistent with observed similar matrix-driven effects in dermal delivery systems.

## 1. Introduction

Oral drug delivery remains the most convenient and preferred route of drug administration for patients; nevertheless, inadequate and highly variable bioavailability continues to present significant challenges to the clinical development of numerous drug candidates, particularly those with poor water solubility [1,2]. In addition to drug solubility, these obstacles are attributable to a complex interaction of physiological, biochemical and anatomical factors within the gastrointestinal (GI) tract, such as enzymatic degradation, fluctuating pH levels, limited epithelial permeability, efflux mechanisms and first-pass metabolism [2]. Consequently, a substantial proportion of orally administered drugs—especially Biopharmaceutics Classification System (BCS) Class II and IV compounds such as curcumin—demonstrate insufficient and unpredictable absorption profiles [3,4].

These limitations can compromise therapeutic efficacy and complicate dose optimization, particularly when sustained systemic exposure or local intestinal effects are required [5]. Therefore, effective formulation strategies must not only enhance solubility but also control drug release, intestinal permeation and barrier interactions.

Formulation optimization is a key strategy to improve drug delivery, bioavailability, therapeutic efficacy and safety. In this context, nanocarrier-based systems have gained increasing relevance for oral administration [2,6,7]. Nanoparticles—typically defined as particles ranging from 1 nm to several hundred nanometers—can encapsulate or bind active pharmaceutical ingredients within their core, matrix, or at their surface. Due to their high surface-area-to-volume ratio, these systems facilitate efficient drug loading and promote improved interactions with biological barriers [8]. Additionally, nanoparticulate drug delivery platforms exhibit advantageous pharmacokinetic profiles, including prolonged circulation time, extended half-life, reduced clearance and increased mean residence time [9]. They may also enhance drug uptake in specific intestinal regions or cell populations through several processes, such as size- and charge-dependent interactions with mucus and epithelial surfaces or through active ligand-mediated receptor targeting [6,10,11].

Lipid nanoparticles (LNPs) have emerged as prominent nanocarrier systems in recent decades, with notable independent contributions from Gasco as well as Müller and Lucks [7,12]. The primary categories of LNPs are solid lipid nanoparticles (SLNs) and nanostructured lipid carriers (NLCs). SLNs were developed to address the limitations associated with traditional colloidal carriers, such as emulsions, liposomes and polymeric nanoparticles, by providing enhanced physical stability, controlled drug release, and capabilities for targeted drug delivery [13,14]. Nevertheless, the highly crystalline lipid matrix structure of SLNs poses challenges, including restricted drug loading capacity and increased risk of drug expulsion during storage resulting from polymorphic lipid transitions [15,16].

NLCs were developed to overcome these limitations by incorporating liquid lipids into a solid lipid matrix [13]. This deliberate disruption of crystal order leads to greater imperfections in the lipid matrix, which enhances drug loading capacity and reduces drug leakage during storage. The inclusion of liquid lipids is believed to create nanocompartments within the solid phase, thereby stabilizing the internal structure and limiting changes in lipid crystallinity over time. Consequently, NLCs provide improved physical stability, decreased drug expulsion, and expanded applicability for both lipophilic and hydrophilic drugs. NLCs have demonstrated versatility across multiple administration routes, including oral, parenteral, pulmonary, ophthalmic, dermal and transdermal delivery [17,18,19,20]. For oral administration, lipid-based systems such as self-emulsifying drug delivery systems (SEDDSs/SMEDDSs), nanoemulsions, SLNs, and NLCs have been widely investigated to improve the bioavailability of poorly soluble drugs by enhancing solubilization, promoting intestinal lymphatic transport and limiting pre-absorptive elimination processes [6,11,21]. Biorelevant digestion and lipolysis models, together with cell-based systems, organoids and in vivo pharmacokinetic studies, have shown that the formulation composition, digestion kinetics, matrix-dependent release, and barrier interactions are assumed to influence supersaturation, precipitation, micellar incorporation and absorption [6,10,20,22]. Thus, the internal matrix architecture and drug distribution within the lipid phase may be regarded as important contributors to oral biopharmaceutical performance, complementing standard physicochemical parameters [6,23].

Although lipid nanoparticle (LNP)-based drug delivery systems show considerable promise, their rational optimization remains challenging. Controlled modification of the lipid matrix to enhance biopharmaceutical efficacy is only feasible if the relationship between the matrix composition and biological performance is clearly understood.

To address this question, we previously conducted a dermal penetration study in which the influence of the lipid matrix composition on biopharmaceutical performance was systematically investigated, showing that an increased solid lipid content led to enhanced long-term dermal penetration compared with nanoemulsions and oil-rich formulations [20]. By consistently employing identical lipid excipients, defined solid-to-liquid lipid ratios and comparable production parameters, we were able to directly compare different lipid nanoparticle types—nanoemulsions, SLNs and NLCs—within an ex vivo porcine skin model [20].

Curcumin was selected as a model compound because it is a natural polyphenolic constituent of *Curcuma longa* with pronounced antioxidant and other biological activities, yet its clinical use is severely limited by extremely low aqueous solubility, poor chemical stability, inadequate tissue distribution and low oral bioavailability [4,24]. These drawbacks make curcumin an ideal probe to study formulation-driven improvements in intestinal permeation and absorption. Lipid nanoparticles, in turn, represent a promising strategy to overcome these limitations and enhance the oral bioavailability of curcumin.

The aim of this study was to determine whether lipid matrix-dependent performance trends previously observed during dermal penetration are conserved under intestinal barrier conditions. For this purpose, curcumin-loaded lipid nanoparticles with identical lipid compositions and production parameters were evaluated in an ex vivo porcine intestinal permeation model to investigate if formulation-related effects can be distinguished from tissue-specific microenvironmental influences [25].

By comparing and evaluating formulation physicochemical properties alongside semi-quantitative permeation metrics, this study aimed to explore possible structure–performance relationships for curcumin-loaded lipid nanoparticles and to assess whether matrix-dependent trends observed in dermal models may also provide insights into intestinal permeation, which could support the future development of oral lipid nanoparticle formulations.

## 2. Materials and Methods

### 2.1. Materials

Curcumin (*Curcuma longa* extract containing 80% curcumin) was selected as a model drug to represent BCS-Class IV compounds (low solubility and low permeability) and was obtained from Receptura Apotheke (Cornelius-Apothekenbetriebs-OHG, Frankfurt, Germany). Cetyl palmitate 15 (CP) served as the solid lipid and Miglyol^®^ 812 (medium-chain triglycerides) as the liquid lipid, and both were supplied by Caesar & Loretz GmbH (Hilden, Germany). Plantacare^®^ 818 (BASF AG, Ludwigshafen, Germany), a C8–C16 alkyl polyglucoside, was used as a surfactant to stabilize the lipid nanoparticles. Purified water produced freshly by a PURELAB Flex 2 water purification system (ELGA LabWater, Veolia Water Technologies GmbH, Celle, Germany) was used as the dispersion medium for all samples.

These formulations, comprising the same solid lipid, liquid lipid, and surfactant, were intentionally replicated from our previous dermal penetration study to allow direct comparison of matrix-dependent effects across skin and intestinal barriers without altering the formulation composition [20].

### 2.2. Methods

**Figure 1:**
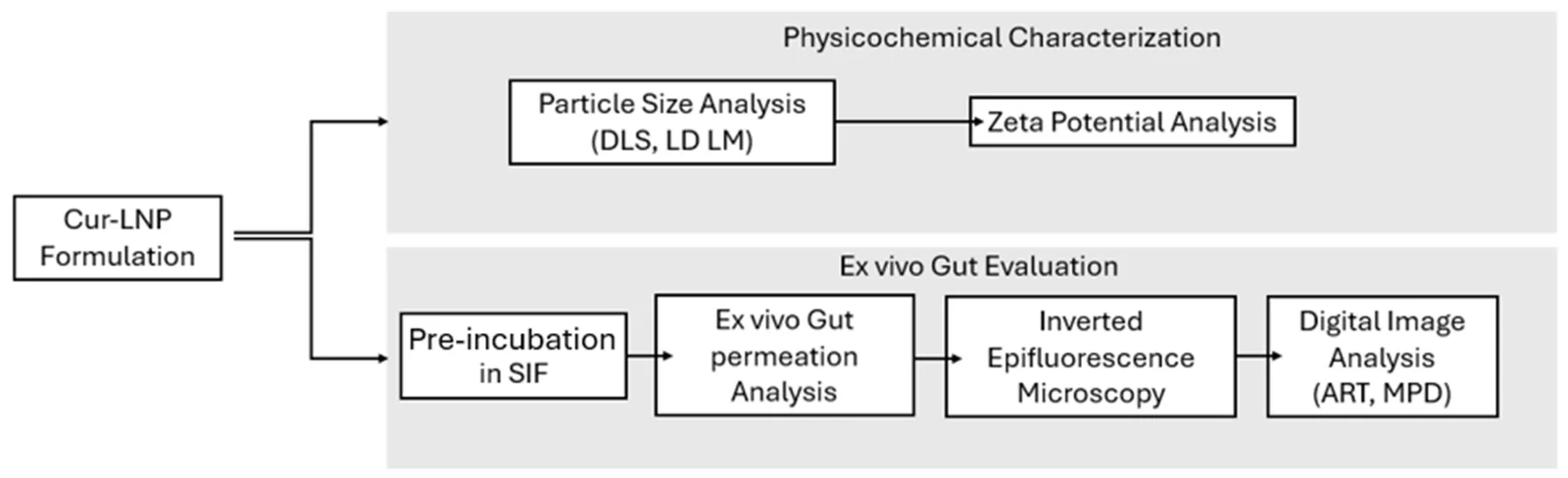
Study workflow illustrating the formulation, physicochemical characterization, and ex vivo evaluation of Cur-LNPs. Abbreviations: DLS, dynamic light scattering; LD, laser diffraction; LM, light microscopy; SIF, simulated intestinal fluid; ART, autofluorescence after RGB threshold; MPD, mean permeation depth.

In the first part of the study, a series of curcumin-loaded LNPs (Cur-LNPs) was prepared that contained different ratios of solid lipid and liquid lipid followed by characterization in terms of particle size, size distribution and zeta potential (Figure 1).

In the second part, the influence of the type of LNP on the intestinal permeability efficacy was evaluated using the ex vivo porcine gut model with subsequent epifluorescence microscopy and digital image analysis [26,27].

#### 2.2.1. Preparation of Lipid Nanoparticles

Cur-LNPs (Table 1) were prepared as previously described [20]. The formulations varied only in the solid-to-liquid lipid ratio, while the total lipid and surfactant concentrations were kept constant. NE contained Miglyol^®^812, SLNs contained cetyl palmitate, and NLCs contained both lipids at CP:Miglyol ratios of 80/20, 60/40, 40/60, or 20/80 (*w*/*w*).

Briefly, the lipid phase and aqueous phase were first prepared separately. For this, the lipid phase consisting of solid lipid and liquid lipid was mixed and melted at 75 °C; meanwhile, curcumin was also added and stirred in the lipid phase until complete dissolution. On the other hand, the aqueous phase consisting of purified water and surfactant was also stirred and heated up to 75 °C. Following this, the aqueous phase was poured into the lipid phase and homogenized using a high-speed homogenizer (Ultra Turrax T25, IKA Werke GmbH & Co. KG, Königswinter, Germany) for 30 s at 25,000 rpm. Afterwards, the mixture was passed through three cycles of high-pressure homogenization using a high-pressure homogenizer (LAB 40, APV Gaulin GmbH/AxFlow GmbH, Düsseldorf, Germany). This process was performed at a temperature of 75 °C while the pressure applied was 500 bar. The formulations obtained were transferred into falcon tubes, cooled in an ice bath and stored at room temperature until further use [28].

#### 2.2.2. Physicochemical Characterization of LNPs

##### Particle Size Analysis of LNPs

The particle size of the Cur-LNPs was analyzed by three different techniques. All three techniques for particle size analysis were performed before and after the process of pre-incubation of the samples as explained in Section 2.2.3 [20].

The particle size and polydispersity index (PDI) of the Cur-LNPs were first determined by dynamic light scattering (DLS) using a Zetasizer Nano ZS (Malvern Panalytical Ltd., Malvern, UK). Samples were diluted with purified water to achieve an appropriate scattering intensity and were measured at 25 °C. The translational diffusion coefficient was obtained from the autocorrelation function of the scattered light intensity and converted to the hydrodynamic diameter (Z-average) using the Stokes–Einstein equation. Measurements were performed in general-purpose mode (software version 7.12) with 10 measurements per sample, and the results are reported as the intensity-weighted Z-average diameter and PDI [20].

As a complementary method, particle size distribution in the micrometer range was analyzed by laser diffraction (LD) using a Mastersizer 3000 equipped with a HydroS dispersion unit (software version 3.72, Malvern Panalytical, Kassel, Germany). This technique was used to detect the potential formation of larger particles resulting from aggregation of Cur-LNPs or the precipitation of free curcumin. Measurements were conducted at a stirring speed of 1750 rpm without sonication, and the data are expressed as the mean of five measurements per sample [29].

Light microscopy (LM) was used as a third, orthogonal technique to visualize larger particles and to verify the LD findings. Samples were examined with an Olympus BX53 microscope (Olympus Deutschland GmbH, Hamburg, Germany) at 200× and 400× magnification.

##### Zeta Potential Analysis

The zeta potential (ZP) of Cur-LNPs was measured by laser Doppler anemometry using a Zetasizer Nano ZS (Malvern Panalytical Ltd., Malvern, UK). This method determines electrophoretic mobility from the frequency shift of scattered light caused by particle movement in an applied electric field. The zeta potential was then calculated from electrophoretic mobility using the Helmholtz–Smoluchowski equation. Measurements were performed at 20 °C in both the original dispersion medium (1% (*w*/*w*) surfactant solution) and purified water adjusted to a conductivity of 50 µS/cm with NaCl solution [22].

#### 2.2.3. Ex Vivo Gut Permeability Study

Ex vivo intestinal tissue penetration of Cur-LNPs was evaluated in a porcine gut model as an indicative readout of intestinal permeation behavior [25]. Fresh porcine small intestine was obtained from a local slaughterhouse and processed within 3–4 h post mortem. For each experiment, a proximal intestinal segment was excised to represent the upper small intestine. This segment corresponded to approximately the first 15–20 cm distal to the pylorus. The segments were opened longitudinally and gently unfolded to expose the mucosal surface while preserving tissue integrity. Major food fragments were carefully removed with tweezers. No additional mechanical scraping or chemical treatment of the mucosa was performed.

**Figure 2:**
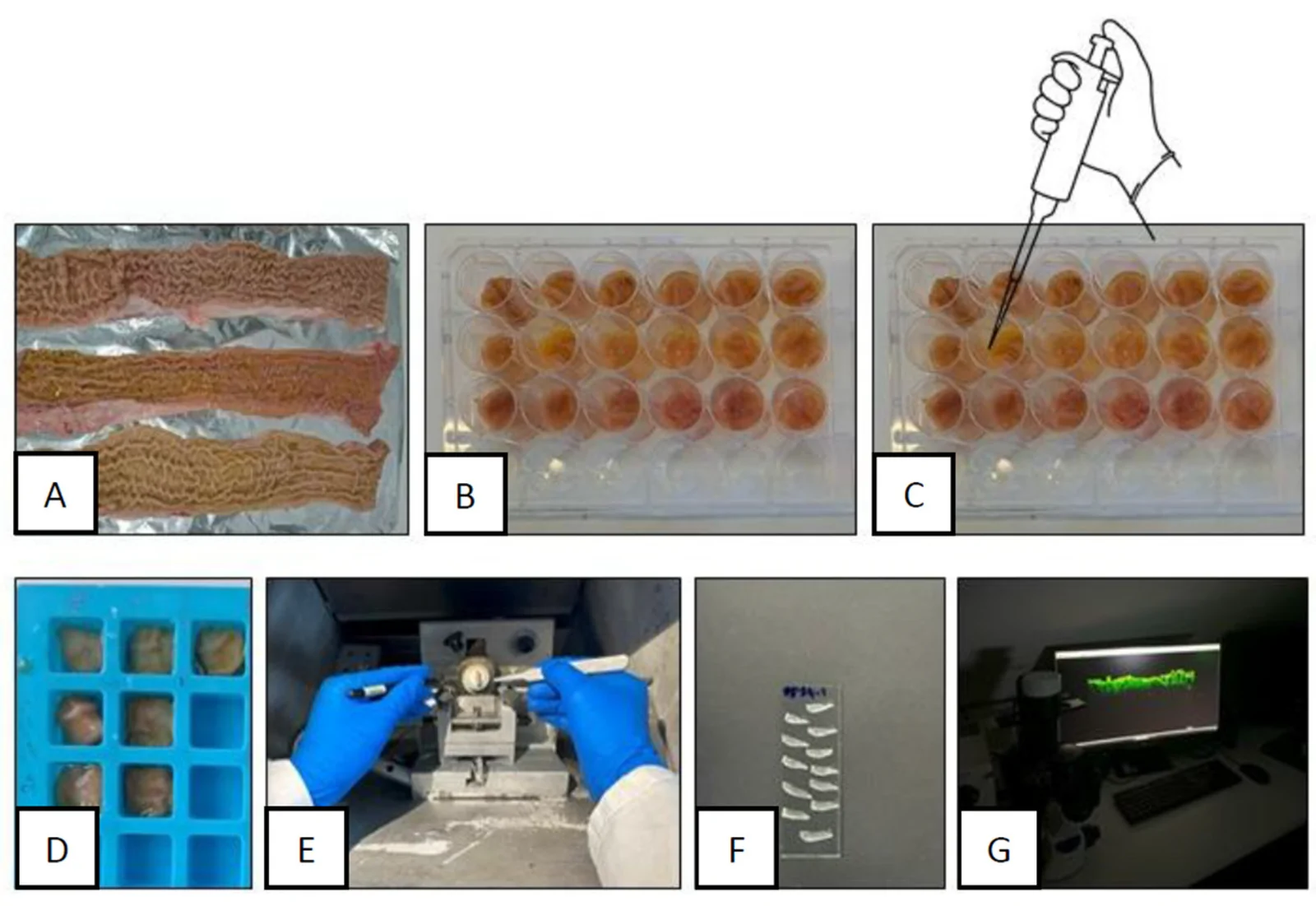
Schematic representation of ex vivo gut permeation studies. (**A**): Fresh porcine intestinal tissue, (**B**): biopsy specimens obtained from porcine intestines, (**C**): application of samples followed by incubation, (**D**): embedding in Tissue-Tek^®^, (**E**): cryosectioning process, (**F**): vertical gut sections mounted on glass slides, (**G**): analysis using epifluorescence microscopy.

Except for this minimal cleaning step, the intestinal tissue was used in its native state, thereby trying to preserve the luminal architecture and mucus layer at the time of sampling. From the gut sections, circular biopsies (15 mm diameter) were carefully punched out and placed individually in each well of a 24-well plate (Figure 2).

Next, 10 µL of each pre-incubated sample (see the Pre-incubation of LNPs Section) was applied to its designated gut biopsy and incubated for either 30 or 60 min. Accordingly, each sample was tested in two separate sets, one incubated for 30 min and the other for 60 min. Untreated biopsies served as controls. After incubation, excess sample was removed by gently washing the biopsies with phosphate-buffered saline (PBS, pH 6.8). The biopsies were then gently dabbed on a cotton towel to remove residual liquid, transferred to molds, and rapidly embedded in Tissue-Tek^®^ (O.C.T.^TM^, Sakura Finetek Europe B.V., Alphen aan den Rijn, The Netherlands). The molds containing the gut biopsies were frozen at −20 °C until further use. All experiments were performed in triplicate, meaning that each sample at each incubation time (30 and 60 min) was tested three times using independent gut sections from three different pigs.

##### Pre-Incubation of LNPs

Before application to the gut biopsies, all Cur-LNPs samples to be tested were pre-incubated in a simplified intestinal medium with surfactant to mimic exposure to an anionic detergent-rich environment. Cur-LNPs were mixed with simulated intestinal fluid (SIF) consisting of 0.01 M phosphate-buffered saline (PBS, pH 6.8) containing 1% *w*/*w* sodium dodecyl sulfate (SDS) while keeping the concentration of curcumin for all samples constant (500 µg/mL) [25]. The mixtures were then stirred at 300 rpm using a magnetic stirrer (universal shaker SM-30 control, Edmund Bühler GmbH, Bodelshausen, Germany) for 15 min. After this, 10 µL samples were applied onto the fresh native gut biopsies using a calibrated pipette (Brand GmbH&Co KG, Wertheim, Germany) (cf. 2.2.3).

##### Visualization by Epifluorescence Microscopy

Curcumin exhibits intrinsic autofluorescence, enabling its direct visualization in intestinal tissue by fluorescence microscopy (FM). Frozen gut biopsies were horizontally sectioned into 40 µm thick slices using a cryomicrotome (Crostat KD-2950, KEDEE^®^, Zhejiang Jinhua, China) [25]. Twelve sections from each biopsy were mounted on glass slides. Samples were analyzed by inverted epifluorescence microscopy (Olympus CKX53, Olympus Deutschland GmbH, Hamburg, Germany), and images were taken with an Olympus DP22 color camera (Olympus Deutschland GmbH, Hamburg, Germany). All images were acquired at 40× magnification using a FITC filter block (excitation 467–498 nm, emission 513–556 nm). The fluorescence light source (130 W U-HGLGPS illumination system, Olympus Deutschland GmbH, Hamburg, Germany) was set to 50% intensity with an exposure time of 50 ms [25,26].

##### Digital Image Analysis

After obtaining the images by FM, they were then subjected to a digital image analysis technique using ImageJ software (version 1.8.0) [30]. Autofluorescence from untreated gut tissue was first subtracted by applying an automated RGB threshold, so that the residual signal in the thresholded images primarily represented tissue-associated curcumin. For each thresholded image, the mean gray value per pixel (MGV/px) was recorded and defined as autofluorescence after RGB thresholding (ART), serving as a semi-quantitative surrogate for the relative amount of curcumin associated with the tissue [20,25,26]. Using the same thresholded images, the vertical extent of the fluorescence signal was measured with the scale function and expressed as the mean permeation depth (MPD), representing the average penetration depth of curcumin-derived fluorescence within the gut section.

#### 2.2.4. Statistical Analysis

The results are presented as the mean ± standard deviation unless stated otherwise. Descriptive statistics and mean comparisons were performed using JASP software (version 0.19.3) [31]. Normality was assessed with the Shapiro-Wilk test and homogeneity of variance with Levene’s test. Normally distributed data were analyzed by one-way ANOVA, with Welch’s correction applied when the variances were unequal [32,33]. Nonparametric data were analyzed using the Kruskal–Wallis test [34]. Where appropriate, Games-Howell, Tukey’s, or Dunn’s tests were used for post hoc comparisons [35]. A *p*-value < 0.05 was considered statistically significant [33].

In the intestinal permeation experiments, each biological replicate consisted of one gut biopsy from a different animal (*n* = 3 pigs per formulation and time point). Each biopsy was sectioned into 12 vertical slices, and three fluorescence images were acquired per slice, yielding 108 images per formulation and time point. These images were treated as technical subsamples within each biological replicate rather than as independent observations. For statistical analysis, the ART and MPD values were averaged across all images and sections from each biopsy, and the resulting biopsy-level means were used for ANOVA or Kruskal-Wallis testing. This approach avoided pseudo-replication by basing statistical inference on the number of animals rather than on individual images or tissue sections.

## 3. Results and Discussion

### 3.1. Preparation and Characterization of Lipid Nanoparticles

**Figure 3:**
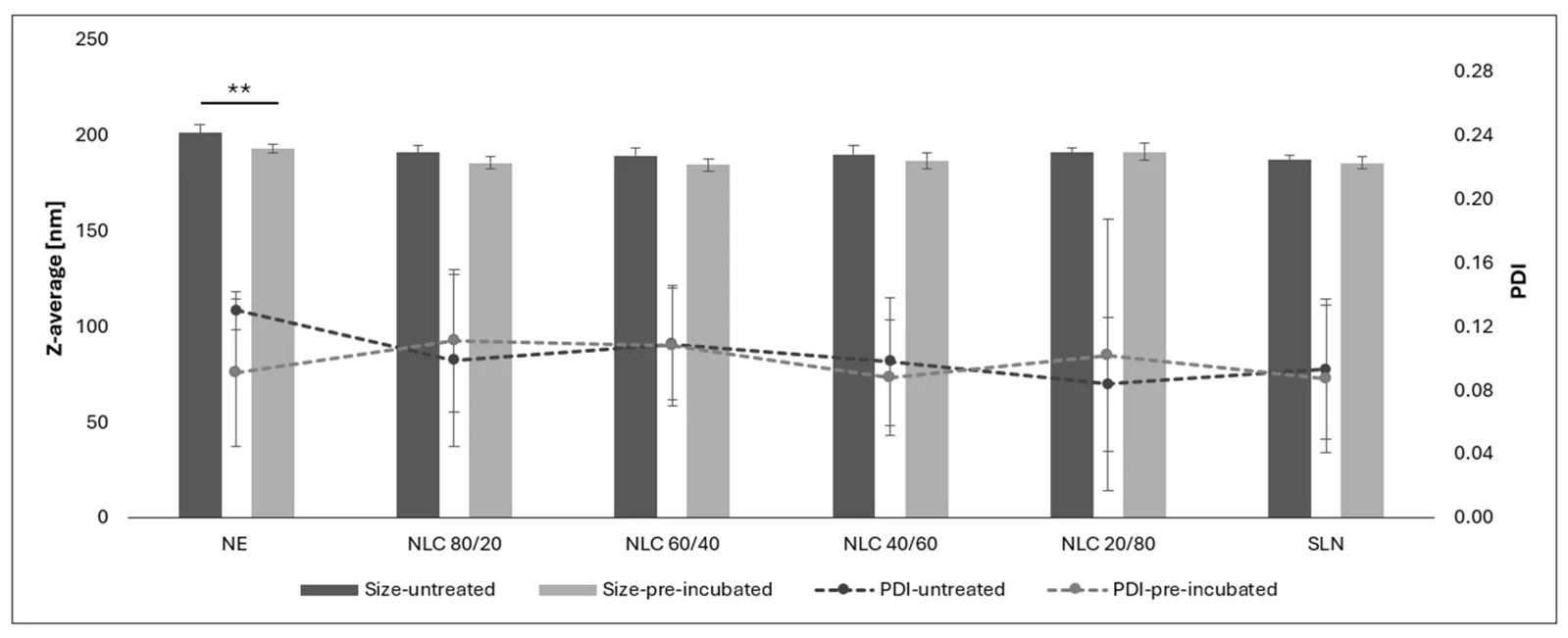
Particle size and PDI of untreated and pre-incubated Cur-LNPs as determined by dynamic light scattering. Data are shown as mean ± SD (** *p* < 0.01).

**Figure 4:**
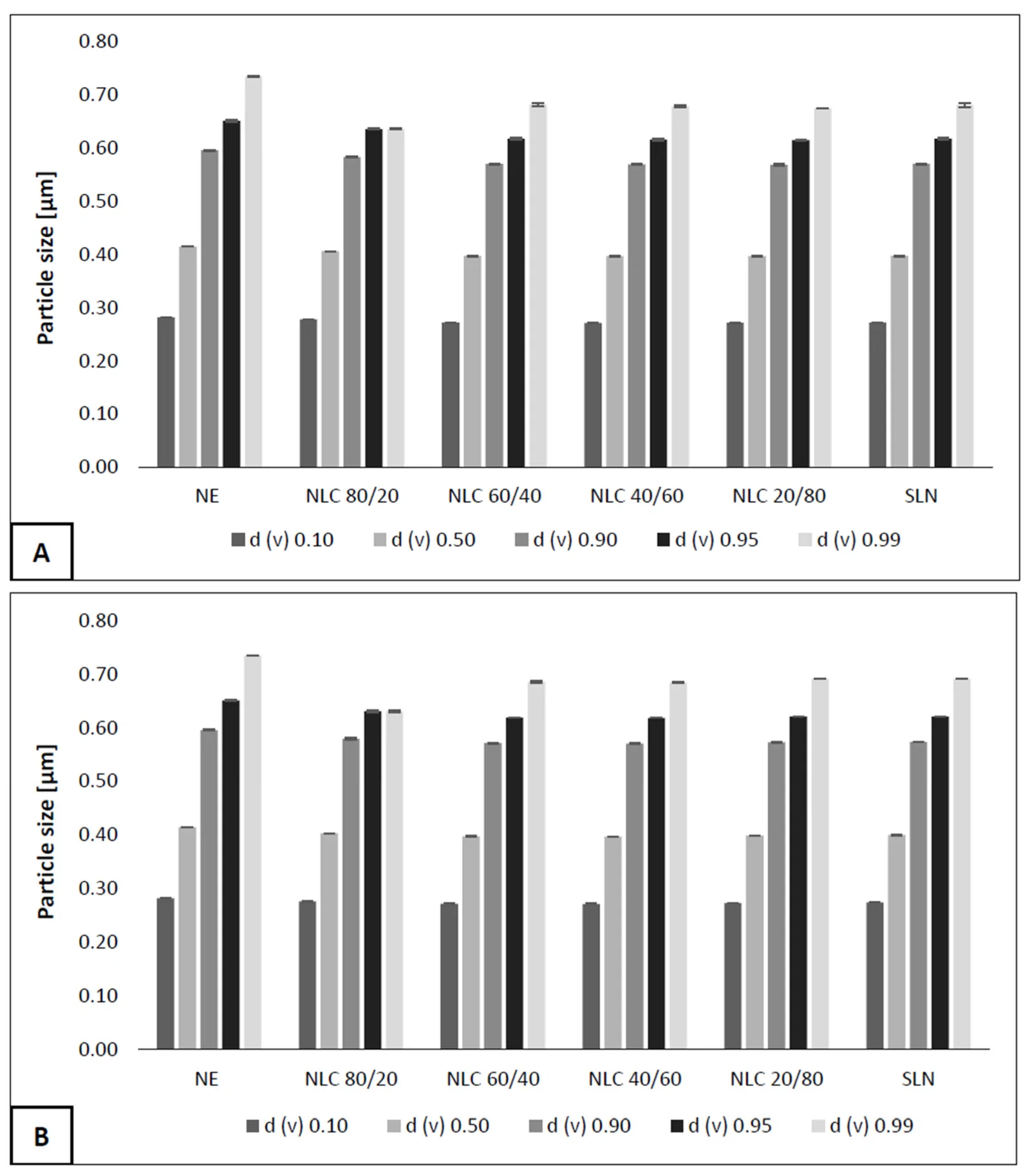
Particle size analysis of Cur-LNPs by laser diffraction. (**A**) Untreated formulations. (**B**) Pre-incubated formulations. Bars represent volume-based particle size distributions. Data are shown as mean ± SD.

**Figure 5:**
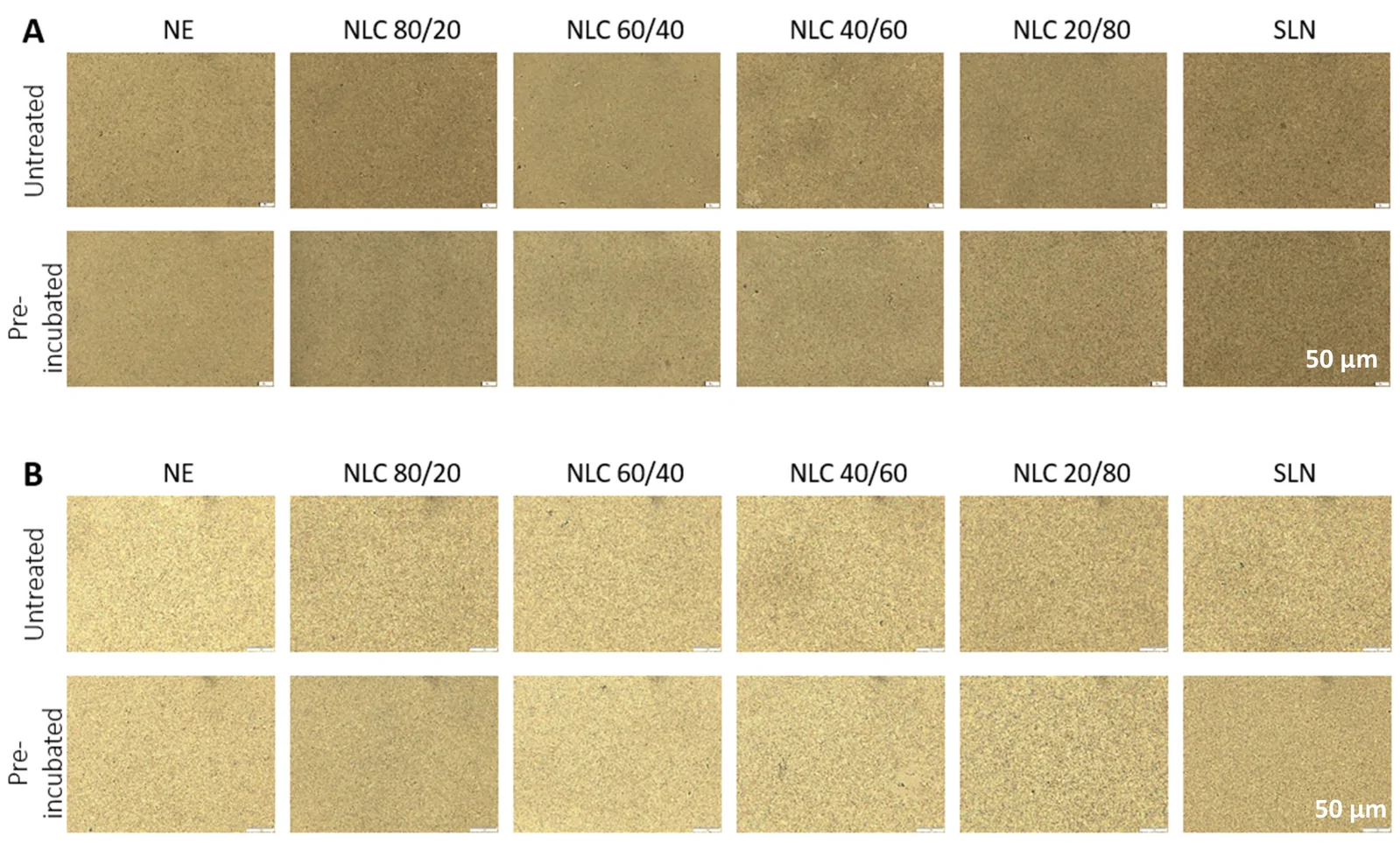
Light microscopy images of untreated and pre-incubated Cur-LNPs. (**A**) 200× magnification. (**B**) 400× magnification. Scale bar: 50 µm.

Cur-LNPs prepared by high-pressure homogenization formed uniform nanoscale dispersions. DLS showed mean particle diameters of about 200 nm for all untreated formulations, with low polydispersity indices (PDI < 0.2) indicating narrow size distributions (Figure 3). LD confirmed the absence of micrometer-sized fractions, as no particles larger than 1 µm were detected based on d(v)0.99 values (Figure 4A). Light microscopy supported these results and showed no consistent presence of large particles or aggregates (Figure 5). At 400× magnification, only occasional larger structures were observed, contributing to less than 1% of the total particle population according to LD estimates.

**Figure 6:**
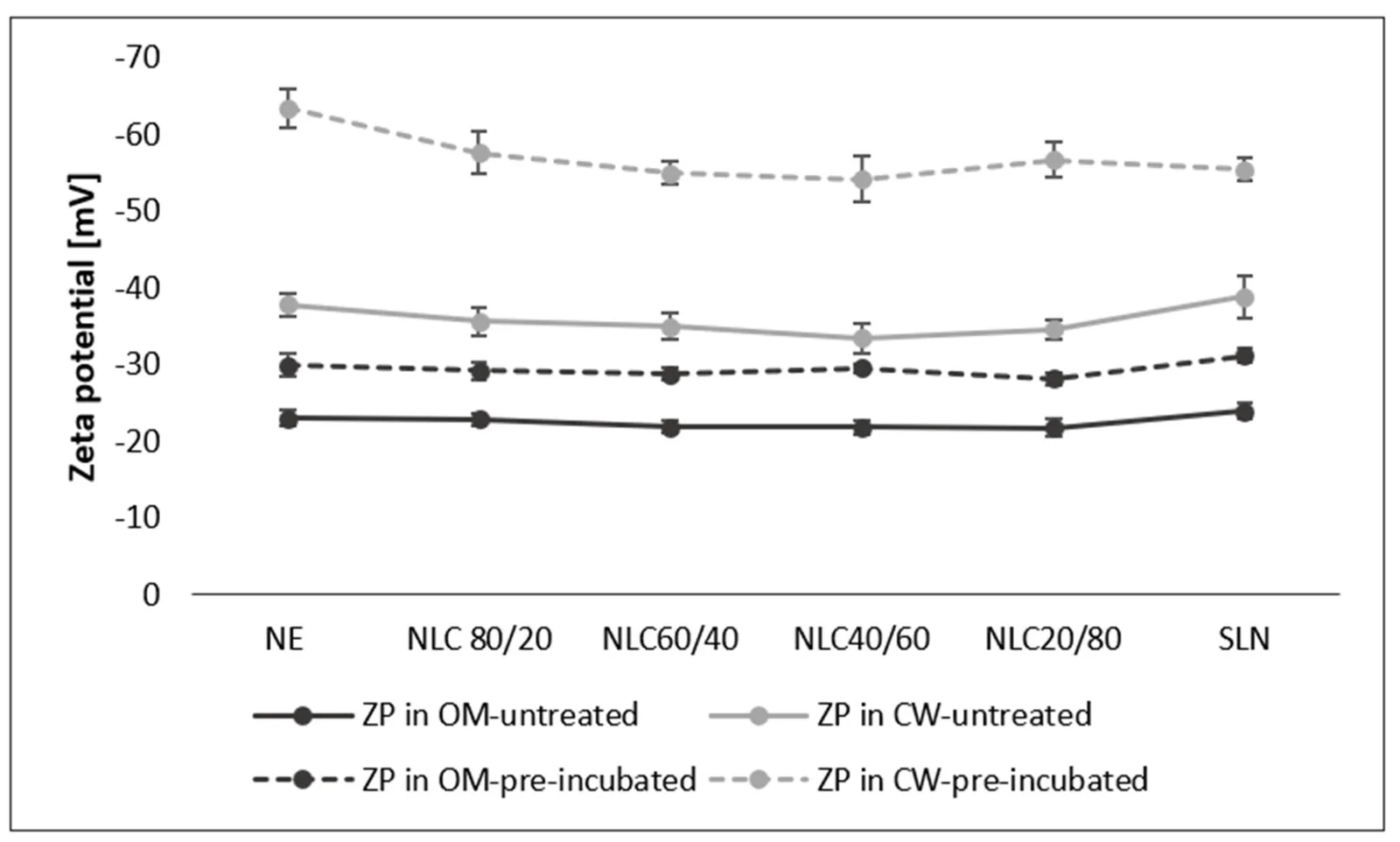
Zeta potential of Cur-LNPs measured in the original dispersion medium (OM) and conductivity-adjusted water (CW; 50 µS/cm). Solid lines: untreated formulations. Dashed lines: pre-incubated formulations.

Across all Cur-LNP types, untreated formulations exhibited moderately negative zeta potentials (approximately −20 to −30 mV in the original medium and −35 to −60 mV in conductivity-adjusted water), with only minor, non-significant differences among NEs, NLCs with varying solid-to-liquid lipid ratios, and SLNs (Figure 6, solid lines). These values were highly reproducible and closely matched those previously reported for this Cur-LNP set, indicating that the two batches are physicochemical comparable and suitable for cross-study comparison [20].

### 3.2. Influence of Pre-Incubation on Physicochemical Properties of LNPs

**Figure 7:**
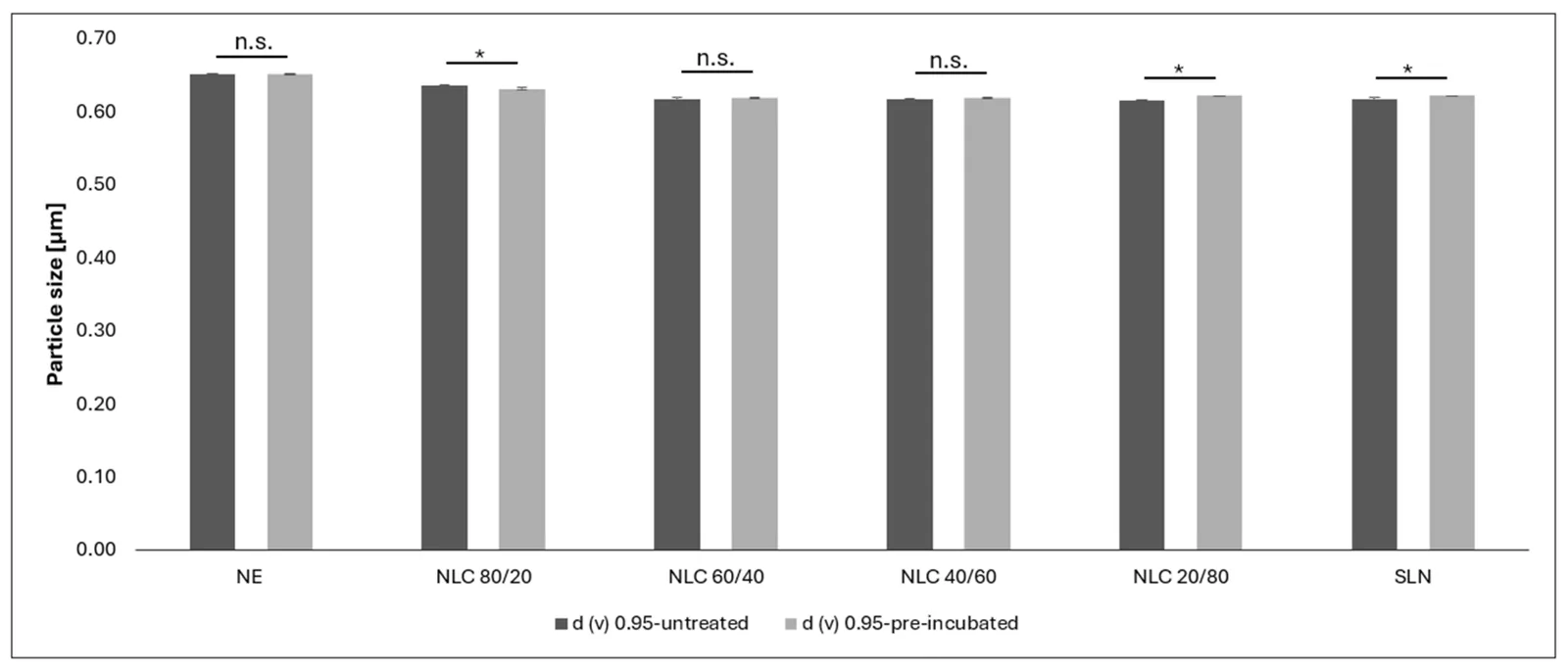
Statistical comparison of LD data (d(v)0.95) of untreated and pre-incubated Cur-LNPs. Data are shown as mean ± SD. (n.s. = not significant, * *p* < 0.05).

Following pre-incubation, all Cur-LNPs formulations retained mean diameters of approximately 190 nm (Figure 3), displayed narrow size distributions (PDI < 0.2; Figure 4B), and showed no formation of larger particles (>1 µm), indicating preserved colloidal integrity under intestinal conditions (Figure 5). Although some formulations exhibited statistically significant yet minor reductions in size (Figure 7), these changes were small in magnitude and did not translate into detectable differences in permeation performance (See Section 3.3). The presence of anionic SDS may promote competitive displacement or the reorganization of surfactants at the oil-water interface, leading to minor alterations in droplet size [36].

After pre-incubation in SDS-containing SIF, all Cur-LNPs shifted consistently toward more negative zeta potentials, largely regardless of matrix composition (Figure 6, dashed lines). This suggests that the post-incubated surface charge is mainly influenced by adsorption or partial replacement of interfacial surfactants by the anionic detergent SDS. Under ex vivo conditions, interactions with negatively charged luminal components may also contribute. In contrast, the untreated values primarily reflect intrinsic, matrix-dependent surface properties.

Biologically, more negative zeta potentials may affect nanoparticle–mucus interactions and epithelial transport [37,38]. A strong negative charge can reduce adhesion to the negatively charged mucus gel but may also weaken nonspecific electrostatic interactions with the epithelial surface [37]. In this ex vivo model, the similar post-incubated zeta potentials of NE, NLCs, and SLNs likely reduce matrix-dependent differences in mucus binding. Thus, the observed permeation profiles are probably driven mainly by drug release and matrix-controlled local concentration gradients rather than charge-mediated retention within luminal mucus.

### 3.3. Evaluation of Gut Permeability of LNPs

**Figure 8:**
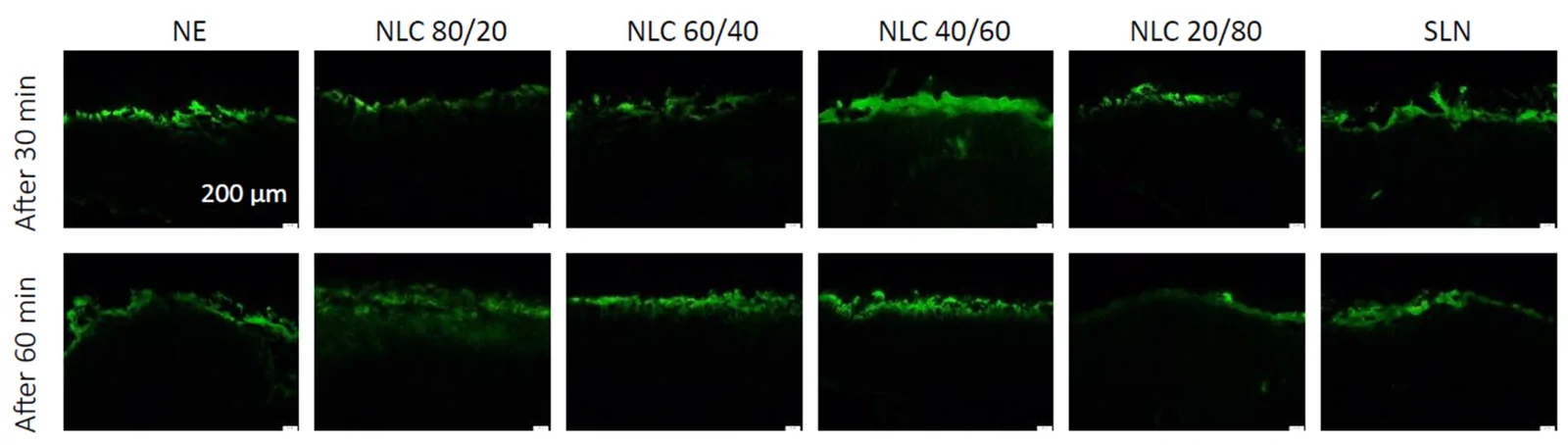
Epifluorescence microscopy images of porcine gut sections following treatment with Cur-LNPs. Upper panel: 30 min post-exposure. Lower panel: 60 min post-exposure. Scale bar: 200 μm.

The intestinal absorption of curcumin from LNPs differed substantially in both the absorbed amount and permeation depth, depending on the formulation type and exposure time (Figure 8). Despite their closely comparable physicochemical characteristics—including particle size, size distribution, and zeta potential—the Cur-LNPs produced clearly distinct, formulation-specific intestinal permeation profiles.

**Figure 9:**
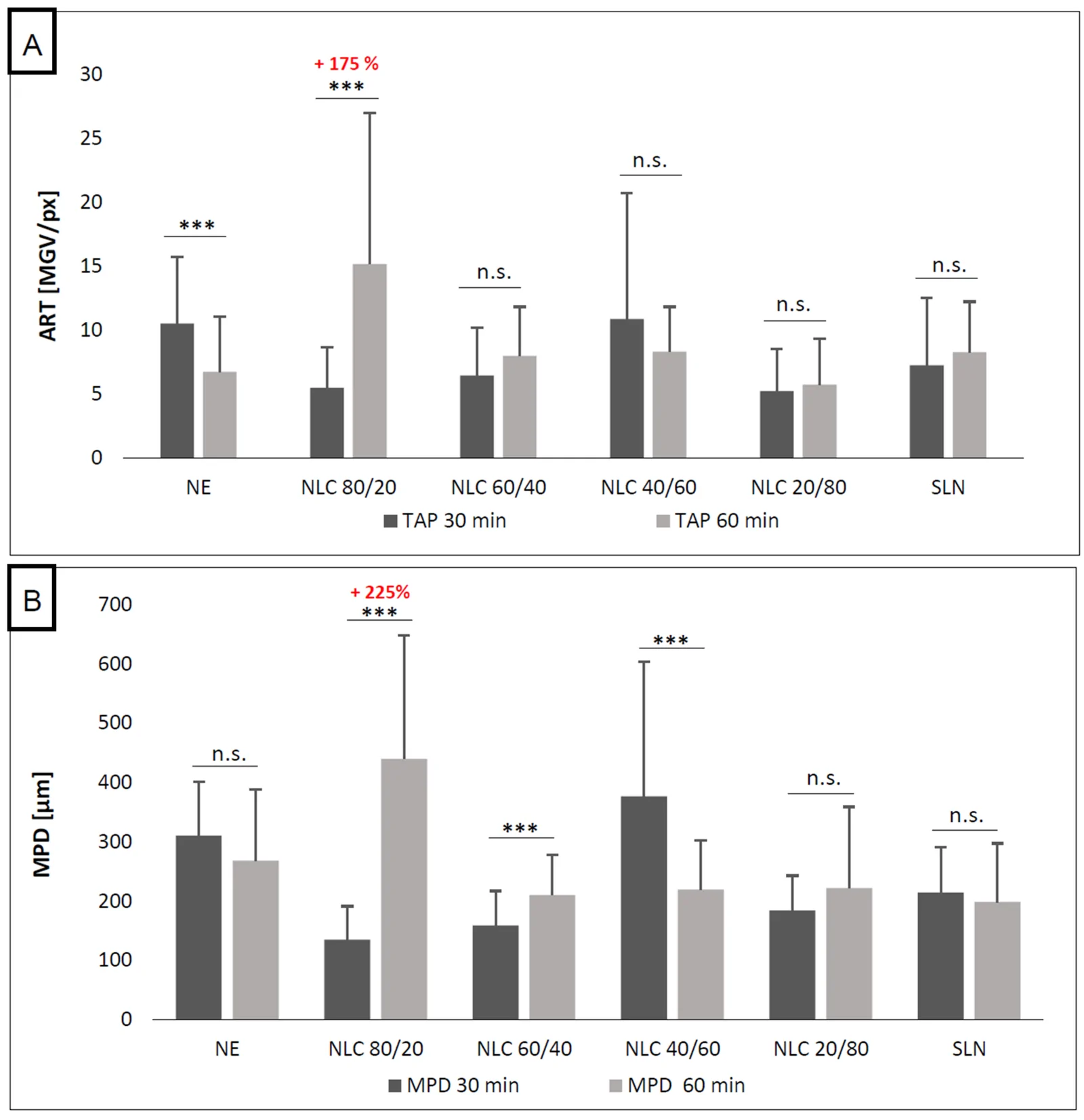
Comparison of short- (30 min, black bars) and long-term (60 min, gray bars) intestinal permeation of Cur-LNPs. (**A**) Semi-quantitative amount of permeated active drug (ART). (**B**) Mean permeation depth (MPD). (n.s. = not significant, *** *p* < 0.001). Data are shown as mean ± SD of *n* = 3 independent biological replicates (gut biopsies from different pigs per formulation and time point).

At the early time point (30 min), NEs and the mixed NLC 40/60 system achieved the highest intestinal permeation, whereas the highly liquid-enriched NLC 80/20 displayed the lowest levels (Figure 9, black bars). This pattern indicates that short-term intestinal penetration is predominantly governed by rapid drug availability at the epithelial surface, consistent with a release-driven process.

Keck et al. reported that nanoemulsions and oil-rich systems provided superior dermal penetration at 2 h, while SLNs performed the worst [20]. Although the relative ranking of formulations is comparable, the underlying mechanisms differ: dermal penetration is mainly controlled by wettability and film formation on the skin surface, whereas intestinal penetration occurs in a hydrated, mucus-rich environment, where transport is dictated by diffusion and convective movement in the luminal fluid.

After an extended 60 min incubation, the intestinal permeation profile changed markedly. NLC 80/20 showed a pronounced increase in both the fluorescence-based permeation readout (ART, +175%) and the image-derived mean permeation depth (+225%), whereas NE exhibited an apparent ~35% decrease in both signals (Figure 9, gray bars). The remaining formulations displayed only minor or no temporal changes, indicating that long-term intestinal permeation characteristics diverge substantially from those observed at early time points.

In the static ex vivo biopsy setup, this pattern is unlikely to indicate a true loss of curcumin after tissue entry. It more likely reflects a decrease in measurable tissue-associated fluorescence over time. Several processes may contribute to this decrease. Curcumin may partially redistribute from the tissue back into the luminal compartment. Additionally, washing and dilution during the PBS rinsing step may further reduce the detectable signal. Furthermore, local degradation or environmental quenching may also affect curcumin fluorescence.

Taken together, the NE data suggest a rapid initial increase in tissue-associated fluorescence. This was followed by a partial loss of detectable signal. This pattern may reflect fast depletion of the donor reservoir. It may also indicate a limited ability to maintain the local concentration gradient at the epithelial surface. However, the ART and MPD should still be interpreted as semi-quantitative surrogates rather than absolute amounts of permeated drug.

A direct comparison of short- and long-term data reveals a distinct temporal progression of formulation performance. Short-term permeation is dominated by rapid, burst-like release and immediate availability of curcumin, favoring NE and oil-rich systems.

By contrast, NLC 80/20 progressively increased both the ART and MPD between 30 and 60 min, indicating a more sustained build-up of tissue-associated curcumin fluorescence and deeper penetration over time (Figure 9). This pattern may be related to a matrix architecture that favors extended release. Such a release behavior could help replenish dissolved curcumin at the epithelial surface. It may also contribute to maintaining the trans-epithelial concentration gradient under the static conditions of the ex vivo model.

From a formulation design perspective, the results indicate distinct intestinal permeation profiles: nanoemulsion produced rapid, burst-like early tissue-associated signals, whereas the flip-flop-type NLC 80/20 showed a more gradual and sustained increase, maintaining elevated fluorescence after 60 min [39]. Thus, the optimal formulation strategy depends on the intended therapeutic profile. This includes whether rapid peak exposure or prolonged tissue exposure is required. The observed differences are most likely related to drug-release behavior and tissue interactions. Bulk physicochemical properties appear to play a smaller role, as they were largely comparable among the curcumin-loaded nanoemulsion, nanostructured lipid carriers, and solid lipid nanoparticles. Release kinetics and matrix crystallinity were not directly quantified. However, this interpretation is consistent with reported lipid nanoparticle matrix behavior. It is also in line with the difference between early and later permeation profiles observed in the present model [20].

Comparison with dermal penetration data further emphasizes the tissue-specific relevance of the lipid matrix effects [20]. Whereas solid lipid-rich formulations, particularly SLNs, markedly enhance dermal penetration over extended durations (+1075%), they provided little additional benefit in the intestinal model and may restrict permeation under hydrated, mucus-covered conditions. Under the static ex vivo conditions applied here, NLC 80/20 therefore seemed to be most suitable for sustained intestinal permeation, while NE favored early permeation. In vivo, however, formulation selection should be guided by the desired pharmacokinetic and pharmacodynamic profile, including the relative importance of C_max_-driven, AUC-driven and target-site exposure-driven effects.

Dermal penetration and intestinal permeation thus follow different temporal trajectories and are governed by distinct microenvironments. In skin, solid lipid-rich matrices initially support diffusion-driven transport, and at later time points, they can modulate the barrier, thereby progressively enhancing penetration [20].

By contrast, intestinal permeation occurs across a hydrated, mucus-covered epithelium and remains strongly dependent on a continuous supply of dissolved drug in the luminal fluid, with the barrier itself undergoing comparatively minor structural changes.

The ex vivo porcine gut model used here represents a static incubation of proximal small intestinal biopsies and therefore only partially reflects the dynamic gastrointestinal environment in vivo. In the in vivo intestinal environment, formulation transport and absorption are influenced by several physiological factors. These include luminal transit, peristalsis, regional differences in surface area and mucus thickness, digestion, pH, and transporter and enzyme expression. Therefore, an administered dose is unlikely to remain at a fixed epithelial site for one hour [40,41].

Accordingly, the 30 and 60 min measurements should be interpreted as time-resolved permeation data. These data were obtained under controlled static ex vivo conditions. They therefore cannot be directly transferred to gastrointestinal transit, regional absorption, or systemic exposure in vivo without further validation.

Porcine intestinal tissue can provide a physiologically relevant translational model. However, species-specific differences may influence drug solubilization, permeation, and metabolism [40]. These differences may involve mucus properties, transporter and enzyme expression, and luminal conditions. The present data should therefore be interpreted mainly as mechanistic evidence of lipid matrix-dependent intestinal permeation. They should not be regarded as quantitative predictors of human oral bioavailability. Future studies could complement these findings with in vitro digestion, cell-based barrier models, and in vivo pharmacokinetic analyses.

Consequently, substantial improvements in intestinal permeation in this ex vivo model are achieved primarily with formulations that provide extended release and sustained local availability, such as NLC 80/20, rather than with the highly solid lipid-rich systems that are advantageous in the dermal context.

### 3.4. Impact of Matrix Composition on Biopharmaceutical Performance of Cur-Loaded LNPs

The biopharmaceutical performance of Cur-loaded LNPs is primarily dictated by the lipid matrix composition rather than by conventional physicochemical readouts. Despite highly similar particle size distributions and zeta potentials, the pronounced differences in intestinal and dermal efficacy demonstrate that the internal matrix architecture is the principal determinant of functional outcome.

Penetration of lipophilic compounds is fundamentally a diffusion-driven process controlled by drug release into the aqueous phase, which constitutes the dominant barrier and rate-limiting step [20]. In accordance with Fick’s first law, maintaining a steep concentration gradient is therefore critical for efficient transport, and matrix structures that modulate drug release and sustain this gradient directly govern penetration performance.

In the present Cur-LNPs series, the solid-to-liquid lipid ratio is hypothesized to affect lipid matrix organization. Based on established lipid nanoparticle concepts, SLNs composed exclusively of solid lipid are expected to exhibit a more ordered crystalline matrix, in which curcumin may be incorporated within a comparatively rigid lipid frame and thus show limited interfacial availability. Conversely, the incorporation of liquid lipid into NLC formulations (80/20, 60/40, 40/60, 20/80) is presumed to increase structural disorder and generate oil-enriched domains, which may accommodate curcumin in more mobile and interfacial accessible regions and thereby favor a more sustained release into the surrounding phase [39].

For flip-flop-type NLCs such as NLC 80/20, previous studies support the assumption that drug molecules may preferentially partition into an oil-enriched outer shell surrounding a more solid lipid core, thereby increasing interfacial drug availability and contributing to sustained release [39,42,43]. Within this mechanistic framework, the increase in intestinal ART and MPD between 30 and 60 min may point to matrix-dependent release behavior. The more sustained permeation profile of NLC 80/20 compared with NE and SLNs supports this interpretation. However, release kinetics and matrix crystallinity were not directly assessed in the present study. This interpretation therefore remains inferential. It should be validated in future studies using dedicated release experiments and solid-state characterization.

We assume that the functional impact of matrix composition is strongly barrier-dependent. In the intestinal model, the aqueous diffusion barrier is preserved by mucus and luminal fluids, so permeation remains strictly governed by release and diffusion into a hydrated environment. Under these conditions, liquid-enriched, structurally flexible matrices such as NLC 80/20 outperform other formulations by maintaining the concentration gradient, as reflected by the marked time-dependent increase in permeation efficacy between 30 and 60 min, whereas NE lose performance once their drug load is exhausted, and highly solidified SLN matrices confer little or no additional benefit. In the dermal model, progressive water loss attenuates the aqueous diffusion barrier and shifts the dominant mechanism toward barrier modulation; here, solid lipid-rich systems such as SLNs show superior long-term performance, consistent with occlusion-driven hydration, reduced transepidermal water loss, and enhanced barrier permeability, with reported penetration enhancements [20].

Dermal penetration and intestinal permeation therefore show distinct temporal profiles. They are also influenced by different tissue microenvironments. In dehydrating dermal tissue, rigid and solid-lipid-rich matrices may initially support diffusion-driven transport. At later time points, they may also affect barrier properties and thereby contribute to increased penetration [20]. In contrast, intestinal permeation occurs across a hydrated, mucus-covered epithelium and depends strongly on the continuous availability of dissolved drug in the luminal fluid, while the epithelial barrier itself is expected to undergo comparatively limited structural change. Under these conditions, flexible and liquid-lipid-rich matrices may show greater external drug availability. This could support sustained diffusion by helping to replenish the local concentration gradient. In contrast, rigid and solid-lipid-rich matrices may retain more drug within the lipid phase. This could limit release into the luminal environment.

These findings suggest that biological performance may not be fully predictable from physicochemical descriptors alone. Matrix structure, drug distribution, and barrier-specific interactions may also play an important role. Time-resolved ex vivo models can help assess these factors. This concept may also be relevant beyond curcumin and the two barrier models investigated here. It could help guide the future development of lipid-based delivery systems.

## 4. Conclusions

Taken together, this study suggests that the lipid matrix composition may contribute to Cur-LNPs performance. This appears particularly relevant when the particle size and zeta potential are closely comparable.

Across the intestinal and dermal ex vivo models, time-dependent differences in penetration were observed. These differences were broadly consistent with matrix-dependent effects on drug release, aqueous partitioning, and local concentration gradients. Liquid-lipid-rich matrices appeared to support more sustained permeation under hydrated intestinal conditions. In contrast, solid-lipid-rich systems were associated with enhanced dermal penetration under dehydration-prone conditions.

Identical Cur-LNPs compositions were evaluated in both models. This comparison may provide mechanistic insight into barrier-specific formulation behavior. It also reduces confounding by formulation design. Nevertheless, the static ex vivo intestinal setup does not fully reproduce gastrointestinal transit, regional barrier heterogeneity, digestion, or species-specific physiology. Therefore, the 30 and 60 min data should be interpreted as controlled, time-resolved ex vivo observations. They should not be directly transferred to human oral bioavailability without further validation.

Overall, these findings suggest that lipid matrix composition may be considered as a barrier- and time-dependent formulation variable. This should complement, rather than replace, conventional physicochemical descriptors. Future work could combine ex vivo permeation with biorelevant digestion and lipolysis assays, release testing, and solid-state characterization. Advanced cell or organoid barrier models and in vivo pharmacokinetic studies could further help validate the proposed matrix-related mechanisms. They may also help assess their translational relevance for poorly soluble drugs.

## Figures and Tables

**Table 1:** Composition of curcumin lipid nanoparticles.

LNP	Curcumin	Miglyol^®^ 812	Cetyl-Palmitate	Plantacare^®^ 818	Water
	% *w*/*w*	% *w*/*w*	% *w*/*w*	% *w*/*w*	% *w*/*w*
NE	0.1	10	0	1	up to 100
NLC80/20	0.1	8	2	1	up to 100
NLC60/40	0.1	6	4	1	up to 100
NLC40/60	0.1	4	6	1	up to 100
NLC20/80	0.1	2	8	1	up to 100
SLN	0.1	0	10	1	up to 100

## Data Availability

The data will be made available upon request.

## Abbreviations

The following abbreviations are used in this manuscript:

ANOVA	Analysis of variance
ART	Autofluorescence after RGB threshold
BCS	Biopharmaceutics Classification System
CP	Cetyl palmitate
Cur	Curcumin
Cur-LNP	Curcumin-loaded lipid nanoparticle
CW	Conductivity adjusted water
DLS	Dynamic light scattering
Dunn’s test	Dunn’s multiple comparison test
FM	Fluorescence microscopy
GI	Gastrointestinal
JASP	Jeffreys’s Amazing Statistics Program
LD	Laser diffraction
LM	Light microscopy
LNP	Lipid nanoparticle
MGV/px	Mean gray value per pixel
MPD	Mean permeation depth
NLC	Nanostructured lipid carrier
NE	Nanoemulsion
NLC 80/20	Nanostructured lipid carrier with 80% liquid lipid and 20% solid lipid
NLC 60/40	Nanostructured lipid carrier with 60% liquid lipid and 40% solid lipid
NLC 40/60	Nanostructured lipid carrier with 40% liquid lipid and 60% solid lipid
NLC 20/80	Nanostructured lipid carrier with 20% liquid lipid and 80% solid lipid
OM	Original medium (1% surfactant solution)
PBS	Phosphate-buffered saline
PDI	Polydispersity index
SDS	Sodium dodecyl sulfate
SD	Standard deviation
SIF	Simulated intestinal fluid
SLN	Solid lipid nanoparticle
ZP	Zeta potential
